# Supplementary material for: Full-Sun observations for identifying the source of the slow solar wind
Source: Nat Commun. 2015 Jan 6;6:5947. doi: 10.1038/ncomms6947 (PMC4354106; doi:10.1038/ncomms6947)
Supplement: Supplementary Information — Supplementary Figure 1 [file ncomms6947-s1.pdf]

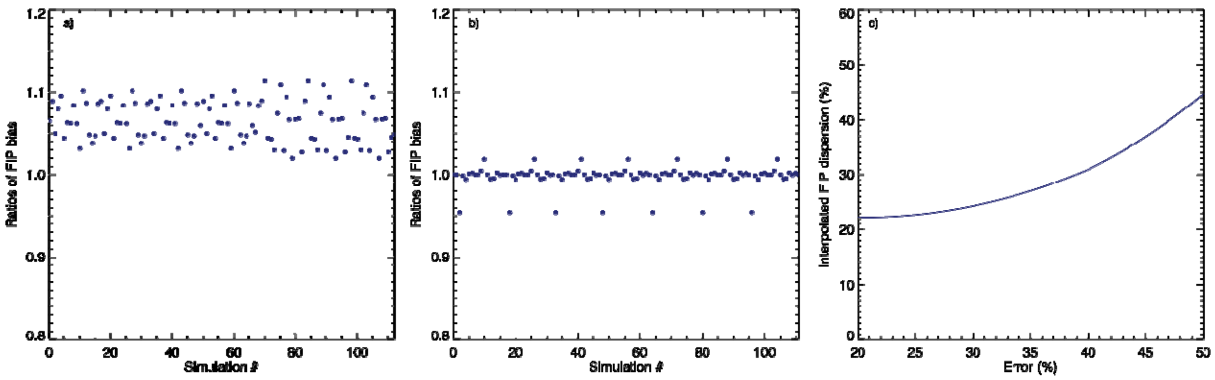

Supplementary Figure 1: Test of the FIP bias measurement method

Exploration of the sensitivity of our FIP bias measurements using a generative model. Left panel: ratio of FIP bias factors calculated from 100 simulations with and without reducing the SW intensities by a factor of 2. Center panel: ratio of FIP bias factors calculated from 100 simulations with and without reducing the Fe abundance by a factor of 2. Right panel: dispersion in FIP bias factors from 100 simulations as a percentage of the average FIP bias factor, plotted as a function of the intensity error.
